# Supplementary material for: Saccharomyces boulardii (CNCM I-745) improves intestinal damage in sepsis by remodeling intestinal flora structure
Source: Front Cell Infect Microbiol. 2025 Jul 31;15:1602792. doi: 10.3389/fcimb.2025.1602792 (PMC12350329; doi:10.3389/fcimb.2025.1602792)
Supplement: Supplementary file 1 [file DataSheet1.zip › summary/summary/8_Advanced_analyse/2_PICRUSt2_result/KEGG/Group.CW0_vs_AW0/PICRUSt2_KO_Group.CW0_vs_AW0_diff.pdf]

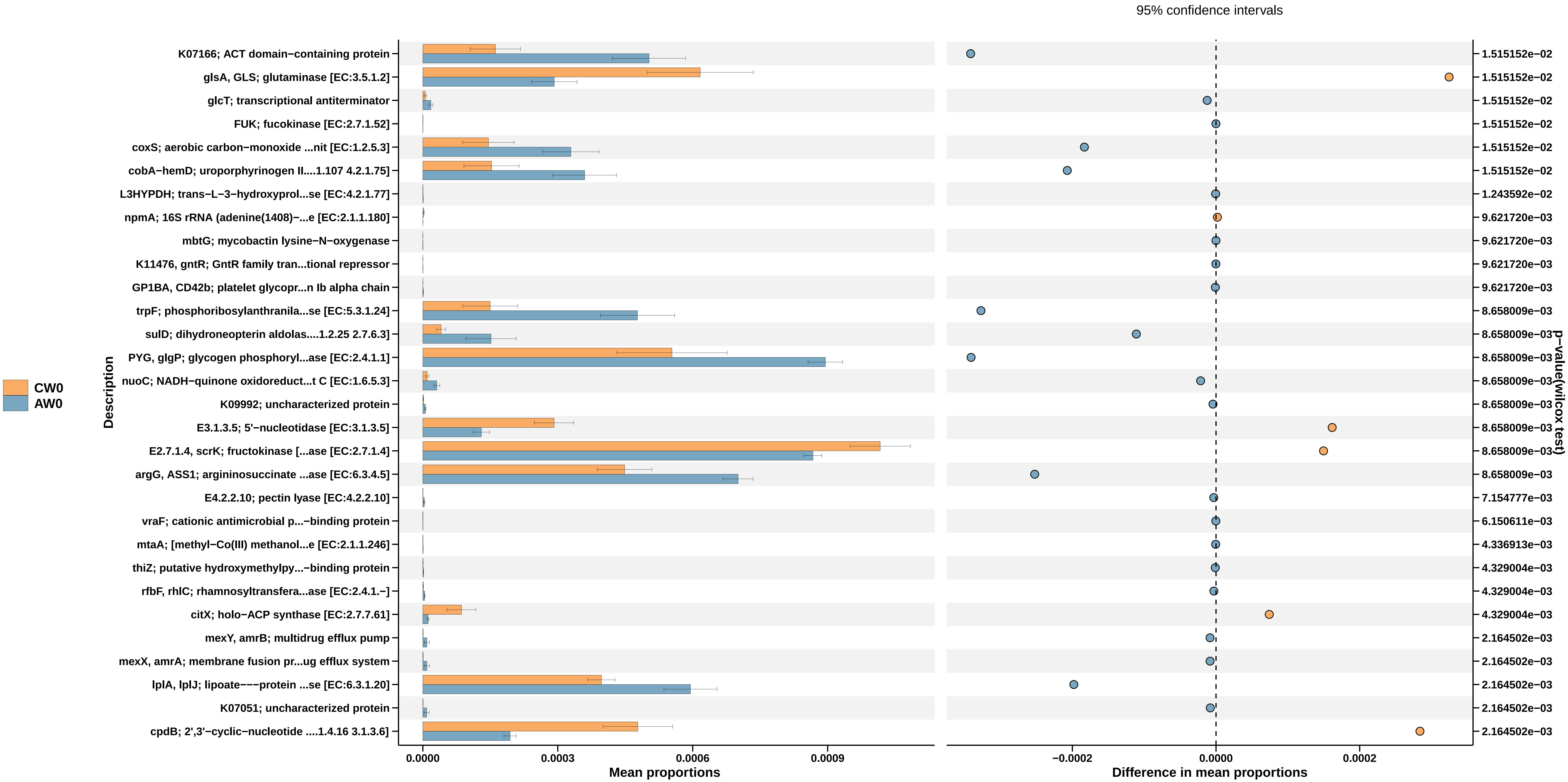
95% confidence intervals
CW0
AW0
Description
K07166; ACT domain−containing protein
glsA, GLS; glutaminase [EC:3.5.1.2]
glcT; transcriptional antiterminator
FUK; fucokinase [EC:2.7.1.52]
coxS; aerobic carbon−monoxide ...nit [EC:1.2.5.3]
cobA−hemD; uroporphyrinogen II....1.107 4.2.1.75]
L3HYPDH; trans−L−3−hydroxyprol...se [EC:4.2.1.77]
npmA; 16S rRNA (adenine(1408)−...e [EC:2.1.1.180]
mbtG; mycobactin lysine−N−oxygenase
K11476, gntR; GntR family tran...tional repressor
GP1BA, CD42b; platelet glycopr...n Ib alpha chain
trpF; phosphoribosylanthranila...se [EC:5.3.1.24]
sulD; dihydroneopterin aldolas....1.2.25 2.7.6.3]
PYG, glgP; glycogen phosphoryl...ase [EC:2.4.1.1]
nuoC; NADH−quinone oxidoreduct...t C [EC:1.6.5.3]
K09992; uncharacterized protein
E3.1.3.5; 5'−nucleotidase [EC:3.1.3.5]
E2.7.1.4, scrK; fructokinase [...ase [EC:2.7.1.4]
argG, ASS1; argininosuccinate ...ase [EC:6.3.4.5]
E4.2.2.10; pectin lyase [EC:4.2.2.10]
vraF; cationic antimicrobial p...−binding protein
mtaA; [methyl−Co(III) methanol...e [EC:2.1.1.246]
thiZ; putative hydroxymethylpy...−binding protein
rfbF, rhlC; rhamnosyltransfera...ase [EC:2.4.1.−]
citX; holo−ACP synthase [EC:2.7.7.61]
mexY, amrB; multidrug efflux pump
mexX, amrA; membrane fusion pr...ug efflux system
lplA, lplJ; lipoate−−−protein ...se [EC:6.3.1.20]
K07051; uncharacterized protein
cpdB; 2',3'−cyclic−nucleotide ....1.4.16 3.1.3.6]
0.0000
0.0003
0.0006
0.0009
Mean proportions
−0.0002
0.0000
0.0002
Difference in mean proportions
1.515152e−02
1.515152e−02
1.515152e−02
1.515152e−02
1.515152e−02
1.515152e−02
1.243592e−02
9.621720e−03
9.621720e−03
9.621720e−03
9.621720e−03
8.658009e−03
8.658009e−03
8.658009e−03
8.658009e−03
8.658009e−03
8.658009e−03
8.658009e−03
8.658009e−03
7.154777e−03
6.150611e−03
4.336913e−03
4.329004e−03
4.329004e−03
4.329004e−03
2.164502e−03
2.164502e−03
2.164502e−03
2.164502e−03
2.164502e−03
p−value(wilcox test)
